# Active surveillance in differentiated thyroid cancer: a strategy applicable to all treatment categories response

**DOI:** 10.3389/fendo.2023.1133958

**Published:** 2023-04-20

**Authors:** Maria Cristina Campopiano, Arianna Ghirri, Alessandro Prete, Loredana Lorusso, Luciana Puleo, Virginia Cappagli, Laura Agate, Valeria Bottici, Sandra Brogioni, Carla Gambale, Elisa Minaldi, Antonio Matrone, Rossella Elisei, Eleonora Molinaro

**Affiliations:** Unit of Endocrinology, Department of Clinical and Experimental Medicine, University of Pisa, Pisa, Italy

**Keywords:** thyroid cancer, active surveillance, rate of growth, recurrence, radioiodine

## Abstract

Currently, the differentiated thyroid cancer (DTC) management is shifted toward a tailored approach based on the estimated risks of recurrence and disease-specific mortality. While the current recommendations on the management of metastatic and progressive DTC are clear and unambiguous, the management of slowly progressive or indeterminate disease varies according to different centers and different physicians. In this context, active surveillance (AS) becomes the main tool for clinicians, allowing them to plan a personalized therapeutic strategy, based on the risk of an unfavorable prognosis, and to avoid unnecessary treatment. This review analyzes the main possible scenarios in treated DTC patients who could take advantage of AS.

## Introduction

The current knowledge on differentiated thyroid cancer (DTC) requires a shift into precision medicine. Closely tailoring medical decisions, treatments, and practices should be based on individualized risk estimates. Furthermore, the last American Thyroid Association guidelines (1) and Italian Expert Consensus (2) for the diagnosis and management of DTC reviewed the traditional *one-size-fits-all* approach, turning it into an individualized management of DTC patients, focused on the estimated risks of recurrence (1) and disease-specific mortality (3). DTC usually has a good outcome with a disease-free survival of approximately 98% and a very low rate of disease-specific mortality (4). Because of this, some years ago, experts have advocated a conservative management approach in selected patients with low-volume tumor burden or slow-progressing DTC (5). Following that, current guidelines (1) have expanded the concept of active surveillance (AS) in DTC management, initially applied mainly to the management of intrathyroidal microcarcinoma (mPTC) (6–11).

As defined by the National Cancer Institute, AS is “a treatment plan that involves closely watching a patient condition but not giving any treatment unless there are changes in test results that show the condition is getting worse” (https://www.cancer.gov/publications/dictionaries/cancer-terms). AS consists of periodical programmed physical examinations, blood tests, and imaging tests that are essential to early detect disease progression and to immediately plan therapies, avoiding either under- or overtreatment. According to this definition, AS is clearly different from both the “watchful waiting” approach, which is a relatively passive follow-up strategy with interventions being triggered by symptoms, and “follow-up care” that involves medical checkups over time in cured patients after treatment, with the purpose of checking disease recurrence (12).

While the management of metastatic and progressive DTC patients is much more clear and requires treatment, the main challenge is to identify the DTC patients (with any grade of persistent disease) who do not need active treatment and could benefit from AS. In some cases, immediate treatment could cause more harm than good, and even it could not be curative. While the current guidelines are available since 2016, in some areas, the management of DTC still diverges from the international indications, and in particular, AS is not considered a feasible and safe strategy (13).

In this review, we discuss the main possible scenarios in treated DTC patients, other than mPTC, who could take advantage of AS. Our objective is to show the relevance of AS in clinical practice, underlining its safety, appropriateness, and effectiveness in selected patients.

## Management of DTC patients not cured by initial treatment

According to the most recent guidelines, DTC (1) patients who did not obtain an excellent response to the initial treatment (i.e., total thyroidectomy *plus* remnant radio-ablation) can be classified into three categories as reported in **Table 1**. These patients should continue with regular checkups but not necessarily have additional treatment. Here, we describe how to tailor the AS according to these categories of DTC patients.

**Table 1:** Response to therapy reclassification in patients with differentiated thyroid cancer treated with total thyroidectomy and radioiodine remnant ablation (1).

Category	Definition
Excellent response	Negative imaging *and* *either* suppressed Tg < 0.2 ng/ml *or* TSH-stimulated Tg < 1 ng/ml
Biochemical incomplete response	Negative imaging *and* Suppressed Tg ≥ 1 ng/ml *or* Stimulated Tg ≥ 10 ng/ml *or* Rising anti-Tg antibody levels
Structural incomplete response	Structural or functional evidence of disease, with any Tg level, with or without anti-Tg antibodies
Indeterminate response	Nonspecific findings on imaging studies, *or* Faint uptake in thyroid bed on RAI scanning, *or* Non stimulated Tg detectable, but <1 ng/ml, *or* Stimulated Tg detectable, but <10 ng/ml, *or* Anti-Tg antibodies stable or declining in the absence of structural or functional disease

Tg, thyroglobulin; TSH, thyrotropine.

### Patients with an indeterminate response to initial treatment

**Figure 1:**
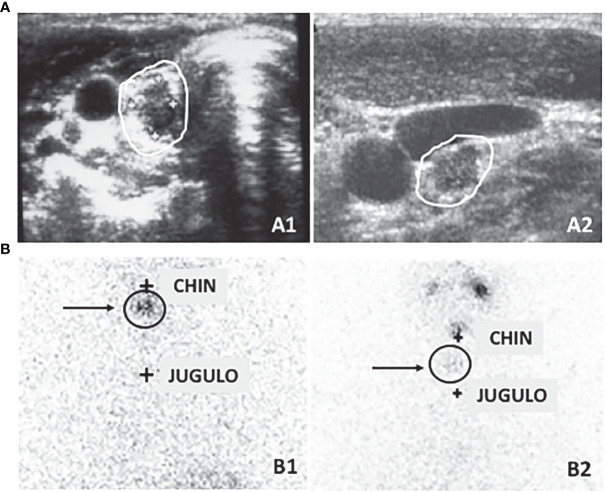
**(A)** Neck ultrasound can detect small lesions that cannot be clearly interpreted as normal or pathological lesions such as sub-centimetric thyroid bed lesion (A1) referable to either thyroid remnant tissue or cancer persistence and indeterminate small cervical lymph nodes (A2). **(B)** Similarly, thyroid bed scintigraphy can detect faint uptake in the thyroid bed that can be due to both a normal remnant or a local persistence or recurrence of the disease (B1) or a small lymph node (B2).

The indeterminate category has biochemical, morphological, or functional findings that physicians could not classify as either absence or persistence of disease (1). Sub-centimetric thyroid bed nodules or indeterminate cervical lymph nodes (**Figure 1A**), faint uptake in the thyroid bed (**Figure 1B**), or non-specific abnormalities on imaging are in this group. Patients with detectable non-stimulated thyroglobulin (Tg) values, but less than 1 ng/ml, stimulated Tg values between 1 and 10 ng/ml, and stable or decreasing Tg antibodies (TgAb)^1^ in the absence of structural disease are also in this category (1).

The prevalence of indeterminate response (IR) after initial treatment ranges from 4% to 30% and is lower in ATA high-risk patients and increases progressively in intermediate- and low-risk patients (14, 15). Patients treated with either total thyroidectomy or lobectomy alone have a similar rate of IR in different series (16, 17). The nonspecific findings either remain stable or resolve during prolonged observation in most patients, without additional treatment (15, 18). No deaths have been notified in patients with IR up to 10 years of follow-up (14, 16, 17, 19), regardless of the type of initial treatment and the ATA risk category. Conversely, only up to 14% of patients with IR to initial treatment was confirmed to have a persistence of disease, either biochemical or structural, after a median of 6–10 years (16, 17, 19, 20), and this probability increased progressively according to initial risk category (14).

Including a multitude of nonspecific findings, highly different from each other, there is no single strategy to manage patients with IR and they require an even more personalized and tailored approach based on the individual characteristics. In this setting, the trend of serum Tg and TgAb over time (21) should be taken into consideration to identify changes in clinical picture. The evaluation of the serum Tg must also take into consideration the TSH values since there is a strict correlation between these two parameters (21). This is not the case for serum TgAb that can be considered a Tg surrogate marker (22), but independent from TSH values.

Neck ultrasound (US) is the main non-invasive tool in the management of these patients, since the major risk of structural recurrence is in the neck. Unfortunately, by definition, the IR category includes cases with neck lymph nodes not clearly malignant but, at the same time, not clearly inflammatory (**Figure 1A**). Most of them could be clinically irrelevant, considering the low prevalence of enlarging nodules, the slow growth rate, and the rarity of local complications (23–25). In these cases, routine fine needle aspiration (FNA) is not appropriate. Nevertheless, it should be considered in lymph nodes greater than 1 cm and increasing in size over a couple of evaluations, especially if, in case of a positive FNA result, a modification in management would be applied (1). Whenever a lymph node FNA is planned, a concomitant measurement of Tg in the washout of the needle used for the procedure should also be performed (26).

In these patients, AS is the only available strategy able to identify the minority of patients requiring further therapies. Once-a-year check including clinical and biochemical (i.e., serum Tg, TgAb, TSH; free T4) evaluations plus a neck US should be performed at least for the first 5 years for those at higher risk of recurrence, and then continued every 18–24 months.

### Patients with a biochemical incomplete response to initial treatment

Abnormal basal or stimulated Tg or elevated values of TgAb without clear evidence of structural disease define the biochemical incomplete response (BIR) after TT and radioiodine remnant ablation (RRA). Up to 18 months after initial therapy, BIR is found in 10%–20% of DTC patients, and, at variance with IR, the prevalence is similar in all ATA risk categories (1).

Up to 70% of these patients reach the excellent response criteria and the clinical remission over time, without any additional treatment. Moreover, approximately 20%–30% maintain a stable Tg level without structural evidence of disease for many years. Only less than 20% develop a structural disease within 5–10 years (14, 15, 19, 27–29). Moreover, the probability of achieving undetectable Tg could depend on initial risk stratification, which is higher in ATA low-risk patients than in ATA intermediate- and high-risk patients (14). Clinical outcome is generally good in these patients and no deaths have been reported over 10 years of follow-up (14, 15, 19, 27–29). These data suggest that patients with BIR after initial treatment can be managed through AS using Tg and TgAb trends and neck US, without additional interventions to determine whether they will spontaneously reach an excellent response to treatment.

During AS, a sustained increasing Tg trend is strongly suggestive of persistent disease, which should require additional evaluation (1, 30). The doubling time (DT) of serum Tg values is also an important prognostic factor, since prolonged Tg DT (>2 years) is associated with a favorable outcome (31). It is worth pointing out that instead of considering an initial elevated Tg (both basal and stimulated) as a specific marker of persistent disease (30), it should be regarded as an alert sign indicating the need for further evaluation in a patient subset, since the predictive positive value of a single basal value of Tg is low (32–34). Similarly, the increase in the TgAb trend is a warning sign, suggesting the possibility of disease persistence (35, 36). Since TgAb usually disappears 3 years after thyroid ablation (22), its persistence for a long period after initial treatment or its progressive rise, confirmed in at least two to three measurements performed during 1 year of follow up, indicates the presence of a Tg source and consequently could reflect the persistence or recurrence of DTC. In this context, it is crucial to note that Tg or TgAb trends are more helpful than a single determination in predicting disease remission or progression (19, 30, 35). Moreover, Tg and TgAb should always be measured simultaneously, using the same methods over time, to ensure comparability (1).

Recently, after the limitation to RRA use and the promotion to lobectomy for selected patients, the role of Tg and TgAb measurements had to be reassessed (1). In these cases, the Tg level closely depends on the volume of residual thyroid tissue, and it is highly variable from person to person. A Tg value able to discriminate residual thyroid tissue from recurrent or persistent DTC has not been established after surgery alone (1, 18, 37), and the stimulated Tg is worthless in these patients (38). Interestingly, most patients who did not undergo RRA experienced the natural fall of both Tg and TgAb over time in different published series (18, 37, 39–43). Ideally, the residual thyroid tissue could sustain the antigenic stimulus, affecting the disappearance of the serum TgAb. The residual thyroid tissue after TT is probably minimal, and it could not maintain the antigenic stimulus, thus determining the loss of TgAb. Moreover, the initial TgAb levels and degree of lymphocytic infiltration could influence the time to TgAb disappearance (42); thus, the role of Tg and TgAb trend, but not the single value, could also be a surrogate marker in non-ablated patients (1, 16, 18, 37). The disappearance process takes time, especially for TgAb, and during this period, patients should be followed up with AS. The observation of a natural decline or stabilization of serum Tg and/or TgAb represents a positive result, while their progressive increase represents an alert sign, and it should prompt further investigations. Furthermore, in patients treated with lobectomy, Tg and TgAb measurement is useless, because we are not able to determine how much of the total Tg and TgAb depend on residual lobe or on persistent disease (1, 18, 37). In this latter group of patients, neck US becomes fundamental during AS for the early identification of both neck lymph node metastases and/or tumoral foci in the unresected lobe.

Also, in patients with BIR, AS is a useful option to reduce overtreatment and, at the same time, to early identify the few real structural recurrences. Periodic evaluations (every 6–12 months during the first 5 years and then every 18–24 months) based on Tg, TgAb measurement, and neck US allow physicians to discern low and stable Tg or TgAb trend to increasing levels. Additional evaluations should be reserved for those patients showing a rise in Tg or TgAb, shown in two or more consecutive measurements over time. DTC patients treated with TT only but not RRA or even with lobectomy had a good prognosis *per se.* The concept of BIR is useless in these cases since these patients should be considered cured until proven otherwise and neck US remains the most informative and sensitive tool in their management.

### Patients with structural incomplete response to initial treatment

Structural incomplete response (SIR) identifies a cohort of DTC patients who have not been cured after initial treatment and have evidence of structural disease either immediately after TT or after RRA as shown by post-therapeutic whole-body scan (ptWBS). SIR includes both patients with biopsy-proven disease and patients with structural or functional metastases assessed on clinical scenarios [i.e., positive ptWBS, positron emission tomography with 2-deoxy-2-fluorine-18fluoro-D-glucose (18FDG-PET), computed tomography (CT) scan, etc.].

Fifty percent to 85% of SIR patients continue to have a persistent disease despite additional therapies (15, 19). Consequently, it confirms the highest risk of disease-specific mortality, which is 11% and 57% for lymph node metastases and distant metastases, respectively. The prevalence of SIR is proportional to ATA risk stratification, being higher in ATA high-risk patients than in ATA intermediate- and low-risk patients (1) in whom it is really very rare (44).

Patients with SIR may be led to further therapies or AS, depending on multiple factors, including the size, location, proximity to vital structures, rate of growth, RAI avidity, and a balance between the risks and the efficacy of therapies. Here, we describe the different possibilities and approaches of AS in SIR patients according to the site of the structural disease.

#### Cervical lymph node metastases

Cervical lymph nodes represent the most common site of persistent and recurrent DTC, occurring in up to 30% of DTC patients and 75% of ATA high-risk patients, especially in those with lymph node metastases at diagnosis (1, 45). Additionally, the number and the size of the involved lymph nodes and the extracapsular extension are risk factors for persistent or recurrent disease (46–48).

**Figure 2:**
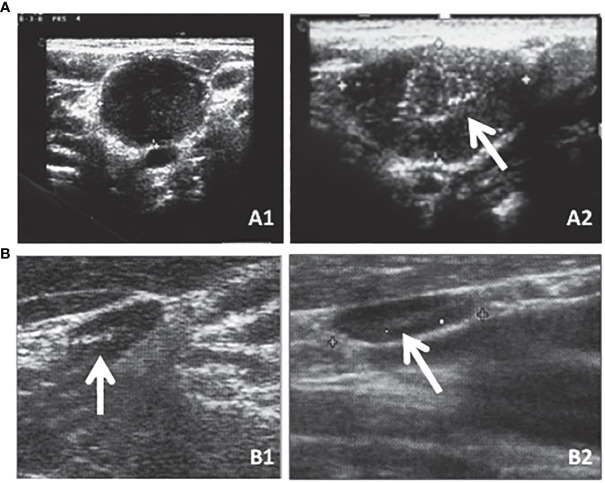
Neck US is the most sensitive tool to distinguish persistent or recurrent lymph node metastases [**(A)** (A1) transversal and (A2) longitudinal sections] that are characterized by well-recognized pathological features (e.g., round shape as shown in A1 or microcalcifications as indicated by the arrow in A2) from enlarged inflammatory lymph nodes [**(B)** (B1) transversal and (B2) longitudinal sections] that are oblong in shape and with an evident hylum as indicated by the arrows in both B1 and B2.

Neck US is the most sensitive tool to distinguish persistent or recurrent lymph node metastases (**Figure 2A**) from enlarged inflammatory lymph nodes (**Figure 2B**) that are characterized by well-recognized pathological features (**Table 2**) (49, 50). In particular, neck US can detect small lymph node metastases in the absence of detectable levels of serum Tg, thus being a fundamental tool in the AS of these patients (38, 51). At the same time, it has been demonstrated that the use of neck US increases the incidence of persistent or recurrent lymph node metastases, likely clinically irrelevant, without changing the disease-specific mortality (4, 52). For this reason, neck US interpretation should not be left to the radiologist but a clinical interpretation should always be performed by the specialist who is following the patient and is aware of their disease status.

**Table 2:** Ultrasound features of normal *vs*. abnormal lymph nodes (49).

Normal lymph nodes	Abnormal lymph nodes
Ovoid shape	Round shape
Normal size	Microcalcifications
Preserved hilum	Cystic components
Absent or hilar vascularization	Increased vascularization
	Increased short axis
	Irregular borders
	Hypoechogenicity

Current ATA guidelines (1) recommend performing neck US to evaluate cervical lymph nodes, and their progression, at 6 to 12 months and then periodically. While resection of large, clinically apparent loco-regional metastases often provides a clinical benefit, it remains unclear whether resection of persistent or recurrent small-volume disease, identified using highly sensitive tools, provides any meaningful clinical benefit. The prevalence of increasing lymph nodes is low, the growth rate is slow as well, and the local complication during AS is rare (53). Moreover, the cure rate after lymph node metastasis re-operation ranges from 20% to 50% in different series, and some cases are required to repeat more than one surgery to achieve disease remission (24, 54–60). Furthermore, up to 30% of patients experience recurrent or persistent lymph node metastases after the second neck dissection (56–58, 60). On the other hand, the surgery failed to remove lymph node metastasis, especially the smallest ones, in up to 10% of patients, despite having biopsy-proven metastases (54, 60). Moreover, up to 15% of patients showed a negative surgical exploration (57, 58). Although neck surgery is relatively safe in expert hands, each surgery carries a significant risk of complications, set as 9% of permanent complications in referral centers (55, 58, 59, 61). Re-operation for recurrent or persistent lymph node metastases is associated with high risks of major and permanent complications, due to the fibrotic tissue and the disruption of the normal anatomic planes after the initial surgery (62).

For all the above-mentioned reasons, abnormal lymph nodes less than 1 cm in the smallest diameter at neck US are candidates for AS and no treatment. Neck US evaluation should be performed every 6–12 months according to the rate of growth, if any, and simultaneously, a biochemical evaluation of TSH, Free T4, and mainly Tg and TgAb is indicated. Routine FNA is not appropriate, but it might be useful in well-proven progressive lymph nodes greater than 1.0–1.5 cm, in which the FNA results, combined with the results of the Tg measurement in the washout of the needle used for FNA (26), will lead to an appropriate and reasonable therapeutic intervention.

#### Distant metastases

Up to 5%–10% of SIR patients may have distant metastasis at diagnosis and 5%–10% may develop distant metastases during follow-up. Almost all distant metastases are in the lungs, while a smaller number is in the bones, brain, and liver. Distant metastases are the most frequent cause of disease-specific mortality, especially in older patients. While 10-year survival rates >95% have been documented in young patients with distant metastases, a median 10-year survival rate of <50% can be expected in older patients with distant metastases (63–66). Ten-year overall survival drops to 10% when distant metastases are not responsive to radioiodine therapy (67) and overall survival is significantly worse in patients with bone or brain metastases (68, 69).

Patients with SIR should be regularly assessed by biochemical and appropriate imaging evaluation to plan a personalized strategy. In this setting, Tg could estimate the tumor burden, and Tg doubling time (DT) may represent a prognostic factor since a short Tg DT (<6 months) is associated with a poor outcome (31). In contrast, an incongruous reduction in serum Tg with no concomitant decrease or with an increase in tumor size could be due to a dedifferentiation of the tumoral cells and suggests a radioiodine refractory disease. Cross-sectional imaging provides the most precise information on tumor burden, proximity to contiguous structures, and tumor growth. DT of distant metastases is also a good prognostic indicator of overall survival in patients with metastatic DTC: a shorter DT correlates with a worse overall survival (70). Positron emission tomography (18FDG) may provide additional prognostic information, since 18FDG-PET positive lesions usually have a more aggressive behavior (71, 72).

Two-thirds of patients with metastatic disease demonstrate substantial uptake of radioiodine, but only 42% of them demonstrate structural resolution of disease, and fewer than 10% demonstrate complete resolution of both biochemical and structural disease (67, 68, 73). Moreover, patients who are not responsive to radioiodine treatment have a poor prognosis and a reduced life expectancy (67). For these patients, the probability of obtaining a remission of disease with further radioiodine treatment is low and other strategies should be evaluated.

Radioiodine refractory metastatic patients require a multi-disciplinary approach, since a myriad of aspects should be assessed to guarantee a tailored and integrated management, based on the high risk of adverse outcome. However, not all patients with structural radioiodine refractory disease require immediate treatment. Patients with asymptomatic, stable, or slowly progressive diseases are candidates for AS since they may not require initiation of therapy until tumors reach a critical volume, patients experience symptoms, or vital structures are involved. Treating a small, stable, and asymptomatic disease could expose patients to treatment’s adverse events without giving them a real prognostic improvement. These patients are candidates for AS with 6-month evaluations including the measurement of serum Tg and calculation of its DT (74), as well as the measurement of TgAb especially for those cases with elevated levels whose trend must be accurately evaluated (74). According to the increasing trend of these serum markers, total body CT scans associated in some cases with 18-FDG-PET should be performed to verify the tumor burden increase (74). Further therapy can be considered when tumor burden becomes clinically significant and tumor progression needs to be documented (70), because the benefit of treatment is demonstrated only in this setting (1, 75–77), and it justifies the potential related adverse events of therapy (78, 79).

In case of stable disease without symptoms, with a slow progression, and without life-threatening lesions, risk about systemic treatment outweighs the benefit and AS can provide a prolonged period of symptom-free disease, without side effects of treatments.

The complexity of these situations and the availability of prospective clinical trials should encourage physicians to refer such patients to tertiary centers with specific expertise.

## Conclusions

In clinical practice, AS is the main tool enabling physicians to optimize therapy planning and prevent side effects from unnecessary treatments. AS can ensure appropriate timing and a personalized approach for an active treatment and, at the same time, avoids unnecessary treatment for patients who would not require additional therapy.

AS is indicated as first-line management for patients with a detectable Tg value or TgAb without structural disease and also for those with small, isolated cervical lymph node metastases, especially in cases previously submitted to nodal compartment resection. AS can also be applied in cases of slowly progressing and asymptomatic distant metastases given that the risk of progression or local invasion is low, and the risk of adverse events outweighs the benefit of treatment.

## Author contributions

All authors contributed to the article and approved the submitted version.

## Footnotes

1 In the same assay over time.
